# Supplementary material for: Clinical characteristics of enteric fever and performance of TUBEX TF IgM test in Indonesian hospitals
Source: PLoS Negl Trop Dis. 2024 Jul 25;18(7):e0011848. doi: 10.1371/journal.pntd.0011848 (PMC11315288; doi:10.1371/journal.pntd.0011848)
Supplement: S1 Table — (DOCX) [file pntd.0011848.s001.docx]

Table S1. Result of confirmatory tests on all enteric fever patients

|  | **Confirmed enteric fever** | **Probable enteric fever** |
| --- | --- | --- |
| **Total Positive Subjects, N** | 54 | 49 |
| **Positive blood culture, N (%)** | 51 (94.4) | 0 (0.0) |
| **Positive molecular assay, N (%)** | 11 (20.4) | 0 (0.0) |
| **Serology** |  |  |
| Seroconversion, N (%) |  |  |
| IgM and IgG seroconversion | 2 (3.7) | 7 (14.3) |
| IgM seroconversion, IgG twofold increase | 0 (0.0) | 0 (0.0) |
| IgM seroconversion, IgG high | 3 (5.6) | 7 (14.3) |
| IgM seroconversion, IgG negative | 1 (1.9) | 0 (0.0) |
| IgG seroconversion, IgM twofold increase | 0 (0.0) | 0 (0.0) |
| IgG seroconversion, IgM high | 0 (0.0) | 2 (4.1) |
| IgG seroconversion, IgM negative | 2 (3.7) | 0 (0.0) |
| Twofold increase, N (%) |  |  |
| IgM and IgG twofold increase | 1 (1.9) | 0 (0.0) |
| IgM twofold increase, IgG > 1.1 | 0 (0.0) | 0 (0.0) |
| IgM twofold increase, IgG negative | 0 (0.0) | 0 (0.0) |
| IgG twofold increase, IgM > 1.1 | 0 (0.0) | 0 (0.0) |
| IgG twofold increase, IgM negative | 0 (0.0) | 0 (0.0) |
| IgM or IgG > 1.1 on baseline data, N (%) |  |  |
| IgM and IgG > 1.1 | 30 (55.6) | 33 (67.3) |
| IgM > 1.1, IgG negative | 2 (3.7) | 0 (0.0) |
| IgG > 1.1, IgM negative | 11 (20.4) | 0 (0.0) |
| Negative, N (%) |  |  |
| IgM and IgG negative | 2 (3.7) | 0 (0.0) |
