# Supplementary material for: Clinical characteristics of enteric fever and performance of TUBEX TF IgM test in Indonesian hospitals
Source: PLoS Negl Trop Dis. 2024 Jul 25;18(7):e0011848. doi: 10.1371/journal.pntd.0011848 (PMC11315288; doi:10.1371/journal.pntd.0011848)
Supplement: S2 Table — (DOCX) [file pntd.0011848.s002.docx]

Table S2. Characteristics of correctly and incorrectly diagnosed enteric fever cases

|  | **Correctly diagnosed as enteric fever** | **Incorrectly diagnosed as enteric fever** |
| --- | --- | --- |
| **Total Positive Subjects, N** | 85 | 138 |
| **Demographics** |  |  |
| Male subjects, N (%) | 39 (45.9)^#^ | 84 (60.9) |
| Age, median (IQR) | 17.3 (7.9 – 23.7) | 23.5 (18.1 – 41.3) |
| Age group, N (%) |  |  |
| 1-5 years | 11 (12.9) | 8 (5.8) |
| 6-10 years | 20 (23.5) | 11 (8.0) |
| 11-17 years | 16 (18.8) | 13 (9.4) |
| 18-25 years | 23 (27.1) | 47 (34.1) |
| 26-40 years | 12 (14.1) | 24 (17.4) |
| 41-98 years | 3 (3.5) | 35 (25.4) |
| **Sign and Symptoms at Enrollment** |  |  |
| Duration of fever, median (IQR) | 7.0 (5.0 – 10.0) | 6.0 (4.0 – 8.0) |
| Gradual onset of fever, N (%) | 53 (62.4)^#^ | 53 (38.4) |
| Type of fever, N (%) |  |  |
| Continuous | 41 (48.2) | 81 (58.7) |
| Remittent | 29 (34.1) | 36 (26.1) |
| Intermittent | 15 (17.6) | 21 (15.2) |
| Anorexia, N (%) | 46 (54.1)^#^ | 44 (31.9) |
| Abdominal pain, N (%) | 35 (41.2)^#^ | 23 (16.7) |
| Nausea, N (%) | 63 (74.1) | 99 (71.7) |
| Headache, N (%) | 41 (48.2)^#^ | 88 (63.8) |
| Vomiting, N (%) | 44 (51.8) | 59 (42.8) |
| Epigastric pain, N (%) | 26 (30.6) | 35 (25.4) |
| Cough, N (%) | 37 (43.5)^#^ | 40 (29.0) |
| Diarrhea, N (%) | 32 (37.6)^#^ | 20 (14.5) |
| Constipation, N (%) | 14 (16.5) | 28 (20.3) |
| **Hematology at Enrollment, Median (IQR)** |  |  |
| Hemoglobin (mg/dL) | 12.5 (11.4 – 13.7) | 13.8 (12.4 – 15.2) |
| Leukocyte (x1,000/mm^3^) | 6.1 (4.5 – 7.9) | 6.1 (4.5 – 8.6) |
| Lymphocyte (%) | 24.4 (16.7 – 34.9) | 20.0 (13.1 – 25.9) |
| Platelets (x1,000/mm^3^) | 137.0 (99.0 – 201.0) | 138.0 (90.7 – 185.5) |

Notes: ^#^significant difference between correctly and incorrectly diagnosed enteric fever
