# Supplementary material for: Clinical characteristics of enteric fever and performance of TUBEX TF IgM test in Indonesian hospitals
Source: PLoS Negl Trop Dis. 2024 Jul 25;18(7):e0011848. doi: 10.1371/journal.pntd.0011848 (PMC11315288; doi:10.1371/journal.pntd.0011848)
Supplement: S3 Table — (DOCX) [file pntd.0011848.s003.docx]

Table S3. Characteristics of cases with confirmed etiologies (n = 1003)

|  | **All enteric fever**^a^ | ***Rickettsia spp.***^b^ | **Dengue**^c^ | **Chikungunya**^d^ | ***Leptospira spp.***^e^ |
| --- | --- | --- | --- | --- | --- |
| **Total Positive Subjects, N** | 103 | 102 | 467 | 38 | 45 |
| **Demographics** |  |  |  |  |  |
| Male subjects, N (%) | 47 (45.6)^*⁰†^ | 68 (66.7)^*^ | 280 (60.0)^†^ | 23 (60.5) | 31 (68.9)^⁰^ |
| Age, median (IQR) | 17.5 (8.1-24.0) | 35.0 (19.8-46.3) | 17.5 (10.7-23.2) | 20.4 (11.5-37.1) | 41.2 (25.7-57.5) |
| Age group, N (%) |  |  |  |  |  |
| 1-5 years | 13 (12.6) | 4 (3.9) | 52 (11.1) | 8 (21.1) | 1 (2.2) |
| 6-10 years | 23 (22.3) | 5 (4.9) | 66 (14.1) | 1 (2.6) | 1 (2.2) |
| 11-17 years | 20 (19.4) | 12 (11.8) | 125 (26.8) | 7 (18.4) | 3 (6.7) |
| 18-25 years | 26 (25.2) | 18 (17.6) | 129 (27.6) | 11 (28.9) | 6 (13.3) |
| 26-40 years | 13 (12.6) | 28 (27.5) | 73 (15.6) | 3 (7.9) | 11 (24.4) |
| 41-98 years | 8 (7.8) | 35 (34.3) | 22 (4.7) | 8 (21.1) | 23 (51.1) |
| **Sign and Symptoms at Enrollment** |  |  |  |  |  |
| Fever present at enrollment, N (%) | 81 (78.6) | 84 (82.4) | 356 (76.2) | 34 (89.5) | 33 (73.3) |
| Duration of fever, median (IQR) | 7.0 (4.0-9.0) | 6.0 (5.0-8.0) | 4.0 (3.0-5.0) | 2.0 (2.0-4.0) | 5.0 (4.0-6.0) |
| Gradual onset of fever, N (%) | 59 (57.3)^*†^ | 31 (30.4)^*^ | 76 (16.3)^†^ | 15 (39.5) | 19 (42.2) |
| Type of fever, N (%) |  |  |  |  |  |
| Continuous | 47 (45.6) | 71 (69.6) | 358 (76.7) | 24 (63.2) | 25 (55.6) |
| Remittent | 34 (33.0) | 19 (18.6) | 65 (13.9) | 9 (23.7) | 17 (37.8) |
| Intermittent | 22 (21.4) | 12 (11.8) | 44 (9.4) | 5 (13.2) | 3 (6.7) |
| Anorexia, N (%) | 51 (49.5)^*†$^ | 33 (32.4)^*^ | 154 (33.0)^†^ | 11 (28.9)^$^ | 15 (33.3) |
| Abdominal pain, N (%) | 40 (38.8)^*⁰†$^ | 22 (21.6)^*^ | 84 (18.0)^†^ | 2 (5.3)^$^ | 8 (17.8)^⁰^ |
| Nausea, N (%) | 74 (71.8) | 73 (71.6) | 341 (73.0) | 25 (65.8) | 35 (77.8) |
| Headache, N (%) | 46 (44.7)^*†^ | 70 (68.6)^*^ | 260 (55.7)^†^ | 18 (47.4) | 24 (53.3) |
| Vomiting, N (%) | 52 (50.5) | 44 (43.1) | 246 (52.7) | 14 (36.8) | 28 (62.2) |
| Epigastric pain, N (%) | 32 (31.1) | 28 (27.5) | 112 (24.0) | 6 (15.8) | 11 (24.4) |
| Cough, N (%) | 48 (46.6)^*†$^ | 24 (23.5)^*^ | 79 (16.9)^†^ | 9 (23.7)^$^ | 15 (33.3) |
| Diarrhea, N (%) | 41 (39.8)^*†$^ | 15 (14.7)^*^ | 48 (10.3)^†^ | 3 (7.9)^$^ | 20 (44.4) |
| Constipation, N (%) | 15 (14.6)^†^ | 19 (18.6) | 22 (4.7)^†^ | 3 (7.9) | 5 (11.1) |
| **Hematology at Enrollment, Median (IQR)** |  |  |  |  |  |
| Hemoglobin (mg/dL) | 12.5 (11.3-13.8) | 14.1 (13.0-15.2) | 13.9 (12.7-15.2) | 13.8 (12.2-15.2) | 13.3 (12.3-14.6) |
| Leukocyte (x1,000/mm^3^) | 6.7 (4.8-8.6) | 6.9 (5.4-8.5) | 3.6 (2.6-4.8) | 7.5 (5.6-10.1) | 10.6 (8.1-14.8) |
| Lymphocyte (%) | 23.6 (16.1-33.7) | 21.0 (14.8-26.0) | 25.5 (17.7-36.0) | 17.0 (12.2-23.0) | 6.9 (4.8-11.5) |
| Lymphocyte count (x1,000/mm^3^) | 1521.0 (1015.5-2135.0) | 1388.5 (990.2-1810.0) | 897.0 (585.9-1282.6) | 1326.0 (621.5-1768.0) | 729.0 (540.3-1102.2) |
| Platelets (x1,000/mm^3^) | 144 (106.9-204.0) | 111.5 (74.6-158.0) | 91.8 (67.0-131.0) | 193.3 (162.5-250.5) | 119.0 (70.3-183.5) |

Notes: *^a^*Enteric fever positive by blood culture or PCR or serology. *^b^Rickettsia* positive by PCR or IFA or serology. *^c^Leptospira* positive by PCR or MAT or serology. ^d^Dengue positive by PCR or NS1 or serology. ^e^Chikungunya positive by PCR or serology. ^*^between enteric fever and *Rickettsia* (p < 0.05), ^⁰^between enteric fever and *Leptospira* (p < 0.05), ^†^between enteric fever and Dengue (p < 0.05), ^$^between enteric fever and Chikungunya (p < 0.05). Not all confirmed cases were analyzed due to the small number of other etiologies.
