# Supplementary material for: Clinical characteristics of enteric fever and performance of TUBEX TF IgM test in Indonesian hospitals
Source: PLoS Negl Trop Dis. 2024 Jul 25;18(7):e0011848. doi: 10.1371/journal.pntd.0011848 (PMC11315288; doi:10.1371/journal.pntd.0011848)
Supplement: S4 Table — (DOCX) [file pntd.0011848.s004.docx]

Table S4. Characteristics of patients with *Salmonella* Typhi and *Salmonella* Paratyphi A

|  | ***Salmonella* Typhi** | ***Salmonella* Paratyphi A** |
| --- | --- | --- |
| **Total Positive Subjects, N** | 39 | 10 |
| **Demographics** |  |  |
| Male subjects, N (%) | 22 (56.4) | 7 (70.0) |
| Age, median (IQR) | 11.1 (6.8-23.8) | 18.8 (9.8-21.8) |
| Age group, N (%) |  |  |
| 1-5 years | 6 (15.4) | 1 (10.0) |
| 6-10 years | 12 (30.8) | 3 (30.0) |
| 11-17 years | 7 (17.9) | 1 (10.0) |
| 18-25 years | 6 (15.4) | 5 (50.0) |
| 26-40 years | 6 (15.4) | 0 (0.0) |
| 41-98 years | 2 (5.1) | 0 (0.0) |
| **Sign and Symptoms at Enrollment** |  |  |
| Fever present at enrollment, N (%) | 30 (76.9) | 7 (70.0) |
| Duration of fever, median (IQR) | 7.0 (5.0-9.3) | 4.0 (3.0-5.3) |
| Gradual onset of fever, N (%) | 22 (56.4) | 5 (50.0) |
| Type of fever, N (%) |  |  |
| Continuous | 20 (51.3) | 4 (40.0) |
| Remittent | 10 (25.6) | 6 (60.0) |
| Intermittent | 9 (23.1) | 0 (0.0) |
| Anorexia, N (%) | 22 (56.4) | 2 (20.0) |
| Abdominal pain, N (%) | 15 (38.5) | 4 (40.0) |
| Nausea, N (%) | 29 (74.4) | 6 (60.0) |
| Headache, N (%) | 16 (41.0) | 6 (60.0) |
| Vomiting, N (%) | 21 (53.8) | 7 (70.0) |
| Epigastric pain, N (%) | 11 (28.2) | 4 (40.0) |
| Cough, N (%) | 16 (41.0) | 4 (40.0) |
| Diarrhea, N (%) | 20 (51.3)^•^ | 0 (0.0) |
| Constipation, N (%) | 8 (20.5) | 2 (20.0) |
| **Hematology at Enrollment, Median (IQR)** |  |  |
| Hemoglobin (mg/dL) | 12.3 (11.0-13.9) | 13.1 (11.9-13.5) |
| Leukocyte (x1,000/mm^3^) | 6.0 (4.9-8.4) | 5.7 (4.2-6.4) |
| Lymphocyte (%) | 21.0 (15.4-33.9) | 21.5 (15.2-36.8) |
| Lymphocyte count (x1,000/mm^3^) | 1422.0 (1015.5-1707.8) | 1198.0 (856.7-1467.4) |
| Platelets (x1,000/mm^3^) | 121.0 (82.0-188.0) | 142.3 (114.5-161.3) |

Notes: ^•^p-value <0.05. Among the 54 confirmed enteric fever cases observed in this study, 39 were *S*. Typhi, 10 were *S*. Paratyphi A, and 5 were non-speciated *Salmonella spp.* (excluded from this table).
