# Supplementary material for: Clinical characteristics of enteric fever and performance of TUBEX TF IgM test in Indonesian hospitals
Source: PLoS Negl Trop Dis. 2024 Jul 25;18(7):e0011848. doi: 10.1371/journal.pntd.0011848 (PMC11315288; doi:10.1371/journal.pntd.0011848)
Supplement: S5 Table — (DOCX) [file pntd.0011848.s005.docx]

Table S5. Sensitivity and specificity of TUBEX TF, acute LPS IgG, and IgM by site

| **Sites** | **TUBEX TF score ≥4, N (%)** | | **TUBEX TF score ≥6, N (%)** | | **ELISA IgM, N (%)** | | **ELISA IgG, N (%)** | |
| --- | --- | --- | --- | --- | --- | --- | --- | --- |
|  | **Sensitivity** | **Specificity** | **Sensitivity** | **Specificity** | **Sensitivity** | **Specificity** | **Sensitivity** | **Specificity** |
| Bandung | 15/15 (100.0) | 21/35 (60.0) | 10/15 (66.7) | 27/35 (77.1) | 17/22 (77.3) | 20/21 (95.2) | 22/22 (100.0) | 17/17 (100.0) |
| Denpasar | 1/1 (100.0) | 0/15 (0.0) | 1/1 (100.0) | 3/15 (20.0) | 1/1 (100.0) | 17/17 (100.0) | 1/1 (100.0) | 7/12 (58.3) |
| Jakarta | 0/0 (0.0) | 1/2 (50.0) | 0/0 (0.0) | 2/2 (100.0) | 0/0 (0.0) | 9/10 (90.0) | 0/0 (0.0) | 6/6 (100.0) |
| Makassar | 4/4 (100.0) | 1/28 (3.6) | 3/4 (75.0) | 6/28 (21.4) | 3/6 (50.0) | 16/16 (100.0) | 5/6 (83.3) | 9/10 (90.0) |
| Semarang | 11/12 (91.7) | 11/23 (47.8) | 9/12 (75.0) | 22/23 (95.7) | 5/13 (38.5) | 15/18 (83.3) | 12/13 (92.3) | 10/14 (71.4) |
| Surabaya | 9/9 (100.0) | 15/25 (60.0) | 6/9 (66.7) | 24/25 (96.0) | 5/11 (45.5) | 19/19 (100.0) | 8/11 (72.7) | 11/13 (84.6) |
| Jogjakarta | 0/0 (0.0) | 3/6 (50.0) | 0/0 (0.0) | 6/6 (100.0) | 1/1 (100.0) | 10/10 (100.0) | 1/1 (100.0) | 2/3 (66.7) |
| **Total** | **40/41 (97.6)** | **52/134 (38.8)** | **29/41 (70.7)** | **90/134 (67.2)** | **32/54 (59.3)** | **106/111 (95.5)** | **49/54 (90.7)** | **62/75 (82.7)** |

Notes: Results are shown as number of positive results/number of samples tested (percentage).
