# Supplementary material for: Clinical characteristics of enteric fever and performance of TUBEX TF IgM test in Indonesian hospitals
Source: PLoS Negl Trop Dis. 2024 Jul 25;18(7):e0011848. doi: 10.1371/journal.pntd.0011848 (PMC11315288; doi:10.1371/journal.pntd.0011848)
Supplement: S6 Table — (DOCX) [file pntd.0011848.s006.docx]

Table S6. Sensitivity of TUBEX TF in confirmed and probable enteric fever

|  | **Confirmed Enteric Fever Sensitivity** | **Probable Enteric Fever Sensitivity** |
| --- | --- | --- |
| **TUBEX TF score ≥4** | 40/41 (97.6) | 31/32 (96.9) |
| **TUBEX TF score ≥6** | 29/41 (70.7) | 20/32 (62.5) |

Notes: Results are shown as number of positive results/number of samples tested (percentage).
