# Supplementary material for: Clinical characteristics of enteric fever and performance of TUBEX TF IgM test in Indonesian hospitals
Source: PLoS Negl Trop Dis. 2024 Jul 25;18(7):e0011848. doi: 10.1371/journal.pntd.0011848 (PMC11315288; doi:10.1371/journal.pntd.0011848)
Supplement: S7 Table — (DOCX) [file pntd.0011848.s007.docx]

Table S7. Sensitivity of TUBEX TF in *S.* Typhi and *S.* Paratyphi cases

|  | **Sensitivity in *Salmonella* Typhi cases** | **Sensitivity in *Salmonella* Paratyphi cases** | **Sensitivity in *Salmonella* spp. cases** |
| --- | --- | --- | --- |
| **TUBEX TF score ≥4** | 30/30 (100) | 8/9 (88.9) | 2/2 (100) |
| **TUBEX TF score ≥6** | 22/30 (73.3) | 6/9 (66.7) | 1/2 (50) |

Notes: Results are shown as number of positive results/number of samples tested (percentage).
