# Supplementary figures and images for: Clinical characteristics of enteric fever and performance of TUBEX TF IgM test in Indonesian hospitals
Source: PLoS Negl Trop Dis. 2024 Jul 25;18(7):e0011848. doi: 10.1371/journal.pntd.0011848 (PMC11315288; doi:10.1371/journal.pntd.0011848)

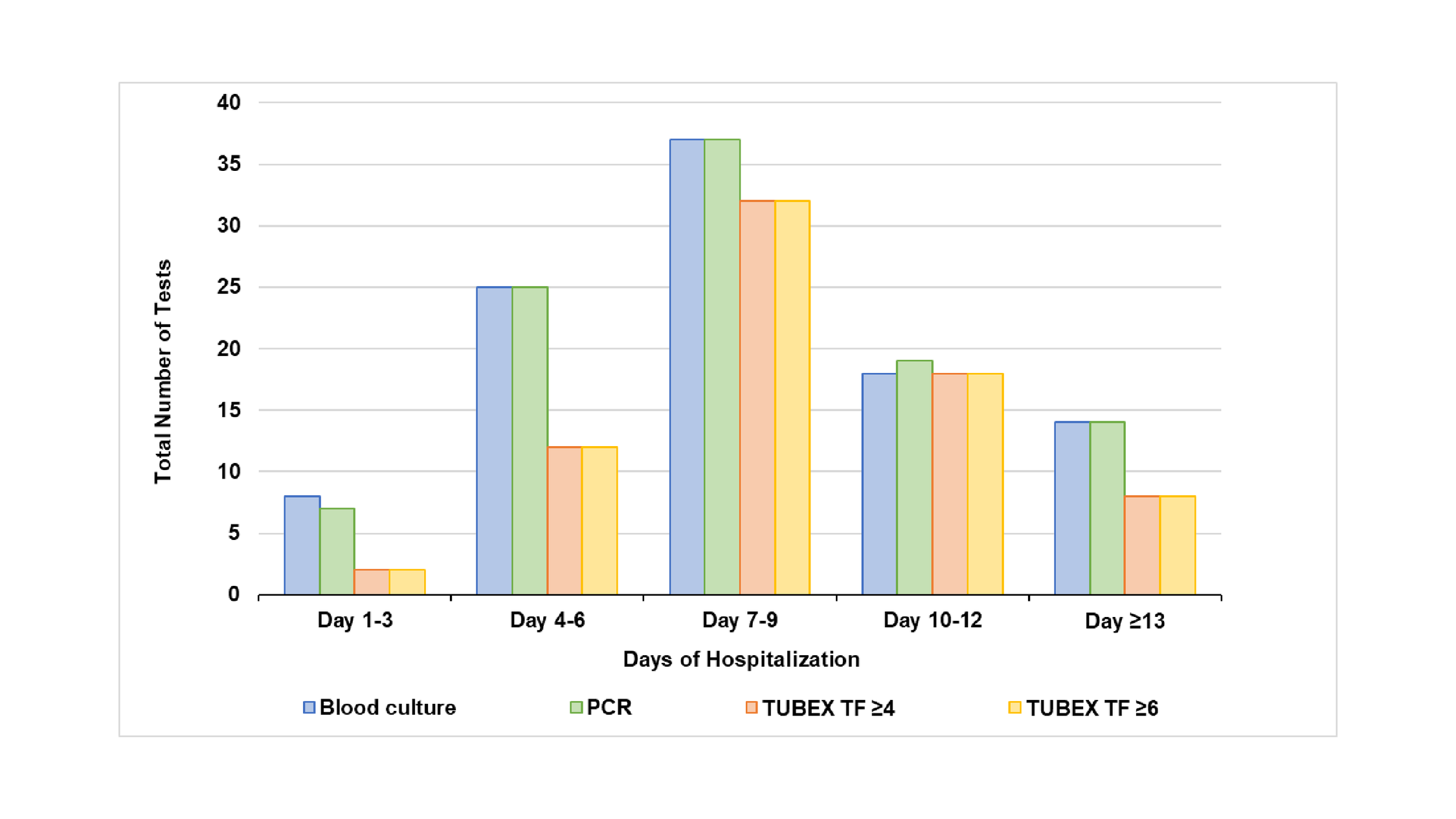

Supplement: S1 Fig — (TIF) [file pntd.0011848.s008.tif]

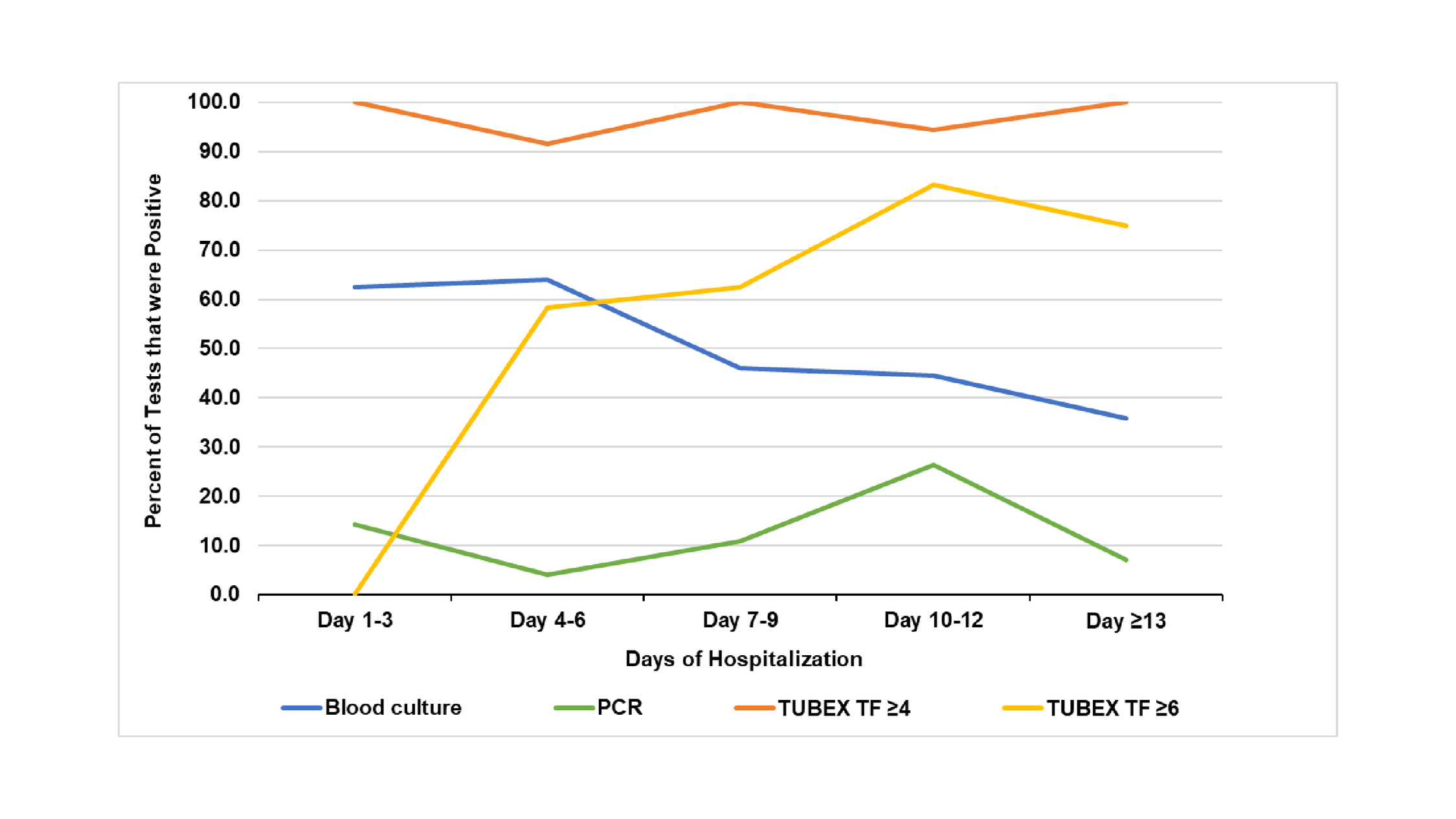

Supplement: S2 Fig — (TIF) [file pntd.0011848.s009.tif]

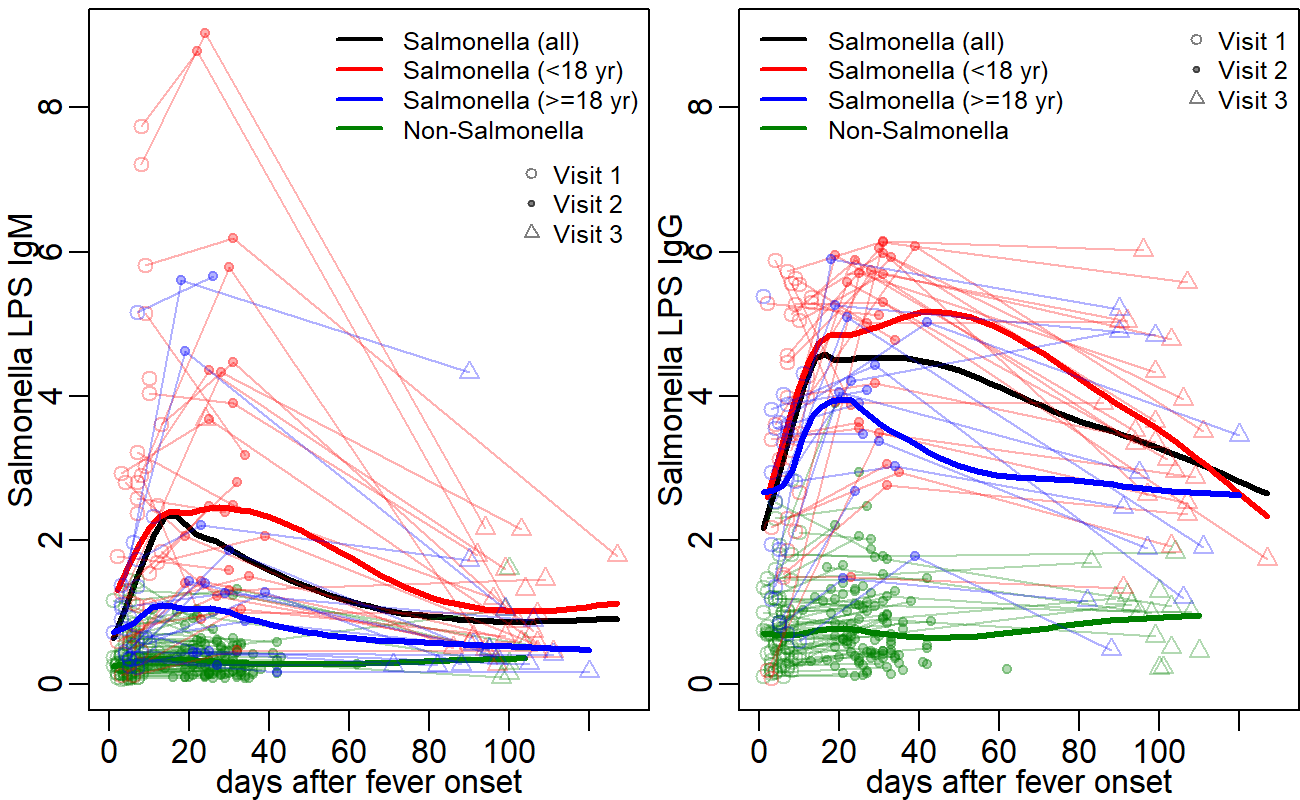

Supplement: S3 Fig — Notes: The figure shows IgM and IgG results for every visit for all subjects. The heavy lines represent the median values of IgM and IgG for all Salmonella cases (black lines), pediatric Salmonella cases (red lines), adult Salmonella cases (blue lines), and all non-Salmonella cases (green lines). A loess smoother was applied in each instance. (TIF) [file pntd.0011848.s010.tif]
