# Supplementary material for: Clinical characteristics of enteric fever and performance of TUBEX TF IgM test in Indonesian hospitals
Source: PLoS Negl Trop Dis. 2024 Jul 25;18(7):e0011848. doi: 10.1371/journal.pntd.0011848 (PMC11315288; doi:10.1371/journal.pntd.0011848)
Supplement: Attachment — Submitted filename: Point-to-point response.docx [file pntd.0011848.s012.docx]

PLOS Neglected Tropical Diseases

March 22^nd^, 2024

Dear Editor,

We thank you for allowing us to submit a revision of our manuscript (PNTD-S-23-02082) entitled “Clinical Characteristics of Typhoid Fever and Performance of TUBEX TF IgM Test in Indonesian Hospitals.”

We thank the reviewers for their reviews and comments, which helped us to improve our manuscript. We have addressed all the comments by the reviewers. Please find our point-by-point response to each comment below. You can find the references at the end of the document. Edits in the manuscript are given in track changes. All authors of this manuscript have seen and approved the content of the revised version.

On behalf of all authors,

Sincerely,

Bachti Alisjahbana

Faculty of Medicine

Universitas Padjadjaran

Bandung, Indonesia

# Response to Reviewers

## Methods

### Reviewer #1:

- line 140, objective mentioned about the performance of Tubex TF only, whereas results shows that performance of Tubex as well as Elisa. Thus, objective should mention about the ELISA

#### Our response:

The authors thank the reviewer for highlighting this point. In this manuscript we analyze the performance of TUBEX TF relative to blood culture, IgM and IgG ELISA serology, and PCR. We have updated the text (lines 142-6) as follows:

*"This study aims to both describe the clinical characteristics of enteric fever patients enrolled in a prospective observational cohort study of patients hospitalized with febrile illness across seven Indonesian cities, and to evaluate the performance of TUBEX TF in a real-world Indonesian hospital setting using blood culture, IgM and IgG ELISA serology, and PCR for Salmonella as reference tests.”*

- study design: appropriate for the study
- sample selection for validity of tubex T and LPS ELISA not mentions is not clearly mentioned in the methods sections

#### Our response:

The authors are grateful to the reviewer for highlighting this crucial point. We have added separate sections for sensitivity and specificity analysis and samples chosen for both analyses (lines 269-92), as follows:

*“Sensitivity analysis*

*Sensitivity analysis of TUBEX TF was performed for all confirmed enteric fever patients who had TUBEX TF testing as part of their hospital evaluation. Sensitivity analysis of IgM and IgG ELISA was performed for all confirmed enteric fever cases where specimens were available for testing at the reference laboratory. Each diagnostic test was additionally assessed with a receiver operating characteristic (ROC) curve, and the area under the curve (AUC) was calculated.*

*Three sensitivity analyses were performed: first, stratification by site to check for heterogeneity of diagnostic accuracy; second, stratification by age (<18 years versus ≥18 years) to assess whether patient age affected diagnostic performance; and third, restriction to only patients with both TUBEX TF and ELISA results to perform a direct comparison of tests.*

*Specificity analysis*

*TUBEX TF specificity was determined from confirmed non-enteric fever cases where TUBEX TF testing was performed based on clinical suspicion. IgM and IgG ELISA specificity was determined from confirmed non-enteric fever cases where samples were available for testing at the reference laboratory. Confirmed non-enteric fever cases in this analysis referred to cases with Rickettsia spp., Dengue, Chikungunya, and Leptospira spp. that were found positive through blood culture or PCR or IFA or MAT. Non-enteric cases with only serology positive were excluded.”*

- what is power of study with available sample size to measure the sensitivity and specificity?

#### Our response:

The authors thank the reviewer for highlighting this point. With the current sample size, we have shown confidence interval achieved in the modified Table 3. For example, sensitivity of TUBEX with cut-off ≥4 is 40/41 (97.6%) with 95% CI: 85.6 – 99.9. As a note we would like to inform you that there is a limitation to the fact that this study was not powered to conduct this type of analysis.

### Reviewer #2:

‒	line 172, in describing the reference laboratory testing, the methodology for the dengue, chikungunya, rickettsia and leptospirosis assays are not described. Would be valuable to include a short description or to reference the MH Gasem et al 2020 paper as the source of that information.

#### Our response:

The authors thank the reviewer for highlighting this point. The text has been revised following the reviewer’s suggestions. We have updated the text (lines 191-7) as follows:

*“Available study samples were sent to a central reference laboratory to be tested at a later date for Salmonella spp. and a variety of other pathogens known to cause acute febrile illnesses. Results were not obtained in real-time and were not used to influence patient care decisions. The methods and testing algorithm for those pathogens, particularly Rickettsia spp., Leptospira spp., dengue virus, and chikungunya virus, have been described previously [Gasem et al. 2020 Dengue tests (PCR, NS1, IgM, and IgG) were performed for all enrolled subjects at the reference laboratory, as described elsewhere [Utama et al. 2019].”*

‒	line 176, it is not clear to me what antigen is used in the IgM and IgG ELISA assay. It would be helpful to know if it is the same as the O:9 lipopolysaccharide antigen used in the TUBEX TF assay.

#### Our response:

The authors thank the reviewer for highlighting this point. The insert kit only stated anti-lipopolysaccharide (LPS). We have also requested further information from MyBioSource and they can only provide membrane protein. The text (lines 197-201) has been updated as follows:

*“Patients who were negative for dengue and other confirmatory tests at study sites were further tested for S. Typhi IgM and IgG antibodies using commercial ELISA kits (MyBiosource Cat. No. MBS580122 and MBS580121) according to the manufacturer’s instructions. Both ELISAs measured antibodies against S. Typhi lipopolysaccharide.”*

‒	line 198, it would be helpful to include the definition of clinical typhoid used

#### Our response:

The authors thank the reviewer for highlighting this point. The text has been revised following the reviewer’s suggestion. We have provided the definition of clinical typhoid, now referred to as enteric fever, as follows (lines 224-7):

*“Clinically diagnosed with enteric fever cases were diagnosed with enteric fever on-site based on clinical presentation and/or initial laboratory examination. Clinically diagnosed with non-enteric fever cases were diagnosed with anything other than enteric fever on-site based on clinical presentation and/or initial laboratory examination.”*

‒	line 201, bring the wording here into line with the wording used in Line 263 in results

#### Our response:

The authors thank the reviewer for highlighting this point. We have harmonized both sentences. The revised lines are in lines 216-20 while the lines below figure 1 (previously line 263) has been removed to avoid redundancy

‒	line 203, suggest outlining with more clarity which participants had these extra tests

#### Our response:

The authors thank the reviewer for highlighting this point. The pathway for tests was previously described in Gasem et al, which is now referenced in the following text (lines 191-6):

*“Available study samples were sent to a central reference laboratory to be tested at a later date for Salmonella spp. and a variety of other pathogens known to cause acute febrile illnesses. Results were not obtained in real-time and were not used to influence patient care decisions. The methods and testing algorithm for those pathogens, particularly Rickettsia spp., Leptospira spp., dengue virus, and chikungunya virus, have been described previously [Gasem et al. 2020].”*

### Reviewer #3:

‒	Although the methods of the AFIRE study from which this study population were drawn have been published previously, some additional detail in the methods on the clinical standard of care for when TUBEX, Salmonella IgG and IgM ELISAs as well as the other diagnostic tests were performed would be helpful. Specifically, the relative timing of blood culture vs TUBEX vs the clinical diagnosis of enteric fever is not entirely clear. Do the clinicians use the blood culture and/or TUBEX results to make the clinical assessment? All the time? Some of the time?

#### Our response:

The authors thank the reviewer for highlighting this point. The following additional details have been included:

*“Both standard of care (SOC) tests and study-specific tests were performed for participants, with SOC tests being performed at the direction of the attending physician in real-time to inform clinical care and study-specific tests being performed at a later time according to a standard study testing algorithm. Initial clinical diagnoses, including enteric fever and non-enteric fever, were made based on clinical judgement alone and later modified based on diagnostic test results.”* (lines 155-61)

*“Blood culture results were immediately provided to the attending physician to inform patient care decisions in accordance with SOC procedures.”* (lines 172-5)

*“Specimens from subjects with clinically suspected enteric fever were tested by TUBEX TF in real-time by hospital laboratory staff at the direction of the attending physician in accordance with SOC procedures. Specimens and accompanying clinical information, including provisional diagnosis, were provided to laboratory staff, and test results were immediately returned to the attending physician to inform patient care decisions.”* (lines 178-82)

*“Available study samples were sent to a central reference laboratory to be tested at a later date for Salmonella spp. and a variety of other pathogens known to cause acute febrile illnesses. Results were not obtained in real-time and were not used to influence patient care decisions.”* (lines 191-4)

‒	Line 180: For the seroconversion—was this based only on the baseline (enrollment sample and the samples collected at 14-28ds (eg not including the 3m samples?) specify in the definition.

#### Our response:

The authors thank the reviewer for highlighting this point. The seroconversion determination used baseline and convalescent samples collected at 14 – 28 days. The following text (lines 204-7) was added:

*“Seroconversion occurred when the index changed from negative in acute samples collected at enrollment to positive in convalescent samples collected during the 14-28 day visit window, or when IgM or IgG increased 2-fold between acute and convalescent samples.”*

‒	Was the differentiation of Typhi vs Paratyphi based on the blood culture/microbiology or was that only done by PCR?

#### Our response:

The authors thank the reviewer for highlighting this point. Typhi and Paratyphi were differentiated based on blood culture and PCR. The following clarifications were added to the text:

*“Blood culture results, including the distinction between S. Typhi and S. Paratyphi, were immediately provided to the attending physician to inform patient care decisions in accordance with SOC procedures.”* (lines 172-5)

*“The TUBEX TF rapid typhoid detection assay (IDL Biotech AB, Karlsbodavägen 39, SE- 168 11 Bromma, Sweden) is a serological test kit that detects IgM antibodies to S. Typhi O:9 lipopolysaccharide antigen. Though S. Paratyphi is also detected due to high antibody cross-reactivity, the test is not able to distinguish the pathogens from each other.”* (lines 182-6)

*“S. Typhi and S. Paratyphi were separately assessed by nested PCR from acute buffy coat specimens from patients diagnosed as non-Dengue and with a positive Salmonella spp. serological assay from acute samples (13, 24).”* (lines 209-11)

‒	Line 185 says that PCR was done for non-Dengue patients with positive serology (is that positive in the acute sample or does that also include patients who seroconverted)? Later in the case definition (Line 200) & Fig 1 Notes, the Confirmed Salmonella cases are defined as positive blood culture AND/OR PCR. Were there any Confirmed cases that were blood culture negative and PCR positive?

#### Our response:

The authors thank the reviewer for highlighting this point. PCR was performed on samples with positive acute serology. There were three cases of negative blood culture and PCR positive. Relevant excerpts from the text are below:

*“S. Typhi and S. Paratyphi were separately assessed by nested PCR from acute buffy coat specimens from patients diagnosed as non-Dengue and with a positive Salmonella spp. serological assay from acute samples (13, 26).”* (lines 209-11)

*“Among the 54 confirmed cases, 43 (79.6%) were confirmed by blood culture only, 3 (5.6%) by molecular detection only, and 8 (14.8%) by both blood culture and molecular detection.”* (lines 334-6)

‒	Since the TUBEX-TF test was developed specifically for S. Typhi, it may be worth at least conducting a sensitivity analysis where the ROC /AUC are calculated for Typhi and Paratyphi separately to determine whether there is any difference in test performance by subtype. At a minimum, the laboratory test results (eg similar to what's included in Fig 1) should be added to Table S3.

#### Our response:

The authors thank the reviewer for highlighting this point. We have added a separate sensitivity analysis of TUBEX TF for *S.* Typhi and *S.* Paratyphi cases in supplemental table 7.

‒	The Data analysis section should include some explanation for why the TUBEX cutoffs of >4 and >6 were chosen.

#### Our response:

The authors thank the reviewer for highlighting this point. The test manufacturer recommends these cut-off values. The text has been clarified as follows:

*“The TUBEX TF assay was performed following the manufacturer’s instructions, where a score of 4 is considered weakly positive and 6-10 considered positive (22).”* (lines 186-8)

*“Two TUBEX TF score categories, ≥4 and ≥6, were assessed since the manufacturer recommends interpreting a score of 4 as weakly positive and 6-10 as positive (22).”* (lines 271-3)

***Minor Comments:***

‒	Line 176: Specify that the antibodies detected by the ELISA kit are to S. Typhi LPS—if more detail is available from the manufacturer about the antigens please include.

#### Our response:

The authors thank the reviewer for highlighting this point. The ELISA kits detect antibodies to *S.* Typhi lipopolysaccharide membrane protein. The test manufacturer is unable to provide more information. The text (lines 200-2) has been updated as follows:

*“Both ELISAs measured antibodies against S. Typhi LPS, which is an antigen known to cross-react with antibodies against S. Paratyphi.”*

‒	Line 215-216: should read: …patient self-report which WAS categorized into sudden OR gradual…

#### Our response:

The authors thank the reviewer for highlighting this point. The text (lines 244-5) has been revised as follows:

*“Timing of fever onset was based on patient self-report which was categorized into sudden or gradual according to patient’s description.”*

--------------------

## Results

### Reviewer #1:

‒	what is dollar sign means in table 1- please mention in line 291-92

#### Our response:

The authors thank the reviewer for highlighting this point. The line has been revised according to reviewer’s suggestion. The dollar sign denotes the comparison between enteric fever and chikungunya. The text below Table 1 now states:

*“Notes: Significant (p < 0.05) differences observed: *between confirmed enteric fever and Rickettsia spp., ^†^between confirmed enteric fever and dengue, and ^$^between confirmed enteric fever and chikungunya. There were no significant differences observed between confirmed enteric fever and Leptospira spp. or confirmed and probable enteric fever.”*

‒	There is discrepancy total salmonella mentioned in table 1 and S3?

#### Our response:

The authors thank the reviewer for highlighting this point. Yes, there is a discrepancy in the total confirmed enteric fever cases in Table 1 and S3 (revised to Table S4). We observed 54 total confirmed enteric fever cases consisting of 39 *S.* Typhi, 10 *S.* Paratyphi A, and 5 non-speciated *Salmonella spp.* The *Salmonella spp.* cases were found through blood culture and could include *S.* Typhi or *S.* Paratyphi A. We decided not to include the 5 cases in Table S4 because we did not know if they were *S.* Typhi or *S.* Paratyphi. The note under Table S4 now states:

*“Among the 54 confirmed enteric fever cases observed in this study, 39 were S. Typhi, 10 were S. Paratyphi A, and 5 were non-speciated Salmonella spp. (excluded from this table).”*

‒	The table 1 is overcrowded, the author could remove the irrelevant information.

#### Our response:

The authors thank the reviewer for highlighting this point. We have reformatted Table 1 to appear less crowded and easier to read.

‒	The analysis plan mentioned about the sensitivity analysis however in result or discussion there is no mentioned about the finding of the sensitivity analysis

#### Our response:

The authors thank the reviewer for their comment. The sensitivity analysis for TUBEX TF and ELISA are stated in the Results section at lines 269-77 and in the Discussion section at lines 510-2 and 515-8.

## Reviewer #2:

‒	line 262-5, does this need to be repeated as in the methods?

#### Our response:

The authors thank the reviewer for highlighting this point. We have deleted these lines to avoid redundancy in this paper.

‒	Table S1, there were relatively few diagnoses on the basis of seroconversion (8+12) or twofold increase in titres (1+0) compared with elevated titres on baseline (43+37). Elevated baseline titres could be due to prior infection or cross-reacting antibodies. This could be commented on in results or discussion.

#### Our response:

The authors thank the reviewer for highlighting this point. Our study actually focus on cases that were confirmed by culture and/or PCR. As you can see in Tables 1 and 2, we divided the confirmed and probable enteric cases. In analyzing accuracy of TUBEX TF and ELISA we only use patients with confirmed enteric cases.

We agree with the reviewer that using serology definition can misidentify prior infection as enteric cases. Therefore, we clarify this limitation with the following statements to the text (line number 313-5): *“Most (75.5%) probable enteric fever cases were identified based on high acute IgM and IgG titers rather than seroconversion or twofold titer increases (Table S1).”*

We also have corrected the statement in Discussion (line 532-8): *“If a single acute specimen is used for diagnosis, it can be difficult to rule-out a previous infection as being responsible for the observed titers. From single baseline specimens in this study, high IgM and/or IgG titers were found in 79.6% of confirmed enteric fever cases and 67.3% of probable cases. Given that serology was the only positive diagnostic test for probable cases, the high titers observed could mislead clinicians toward an incorrect diagnosis if the titers resulted from a previous infection and not the current episode of illness.”*

‒	SF1, in these figures showing the kinetics of the IgM and IgG serology over the three sample points, how were the solid summary lines derived?

#### Our response:

The authors thank the reviewer for highlighting this point. This figure has been changed into SF3 based on the main manuscript’s flow. The solid summary lines are the means of data.

## Reviewer #3:

The analysis generally matches the analytical plan. The results are presented accordingly, although because the different analyses include different patient sets, it is sometimes difficult to follow without repeatedly referring to the methods – particularly in the serologic testing section. There are also some discrepancies in the numbers between what is reported in the text and the various tables/figures that either need better explanation for the differences or need to be confirmed.

#### Our response:

The authors thank the reviewer for highlighting this point. We have added further explanations about the numbers on tables/figures on a separate part on the Methods. We have also tried to rearrange the results based on the numbers of subjects used in the analysis. We hope that this revised version is more simple and easier to understand.

‒	I am also confused by the choice to use the term ‘typhoid fever’ to refer to both S. Typhi and Paratyphi – it would be more accurate to refer to ‘enteric fever’. Similarly, the term ‘confirmed Salmonella’ is imprecise as there are many species of Salmonella; again ‘confirmed/probable enteric fever’ would serve.

#### Our response:

The authors thank the reviewer for highlighting this important point. We have revised the manuscript throughout to use the term ‘enteric fever.’

‒	The data analysis section of the methods section refers to a sensitivity analysis restricted to patients with both TUBEX and ELISA results – it is unclear where this is presented in the Results section.

#### Our response:

The authors thank the reviewer for highlighting this point. Not all samples were tested by both TUBEX TF and ELISA; therefore, the denominators for sensitivity analysis were different for TUBEX TF and ELISA IgG/M. TUBEX TF was conducted by hospital sites and total tested was 41. Meanwhile, ELISA tests were performed on collected samples in the reference laboratory with the available samples were 111 IgM and 75 IgG. This statement has been added to the notes section of Table 3.

‒	Line 254: Provide some explanation for why the 22 subjects were not eligible.

#### Our response:

The authors thank the reviewer for highlighting this point. The following explanation has been added to the text (lines 308-10):

*“Of 1,486 subjects enrolled in the AFIRE study, samples from 22 participants were excluded due to mismatched specimens from one of our sites and 1,464 were eligible for this analysis (Figure 1).”*

‒	Fig 1—why are the undiagnosed that are positive for Serology IgM or IgG not considered probable Salmonella?

#### Our response:

The authors thank the reviewer for highlighting this point. We mistyped the definition for probable enteric fever cases. The probable cases were defined as positive *Salmonella* IgM and IgG antibodies (line 230). The unknown cases only had positive serology IgM or IgG, not both. Due to this, the undiagnosed cases were not included in the probable cases.

‒	Line 269-270: What was the clinician diagnosis for the patients subsequently determined to have Salmonella?

#### Our response:

The authors thank the reviewer for highlighting this point. We have added the statements as followed (lines 325-31):

*“Among cases that were clinically diagnosed as non-enteric fever but later confirmed to be enteric fever, 2 were misdiagnosed as dengue fever, 1 as appendicitis, and 1 as acute limb ischemia. Among cases that were clinically diagnosed as non-enteric fever but later categorized as probable enteric fever, 3 patients were originally diagnosed with gastroenteritis, 3 with dengue fever, 2 with pneumonia, 1 with aseptic meningitis, 1 with viral infection, 1 with urinary tract infection and lung tuberculosis, 1 pharyngitis, 1 fever unknown, and 1 with Hirschprung’s disease and cellulitis.”*

‒	Line 278: specify – antibody indices at baseline.

#### Our response:

The authors thank the reviewer for highlighting this point. The text has been revised according to reviewer’s suggestion. The revised line is in line 339.

‒	Table 1 presents the clinical characteristics of physician diagnosed typhoid cases and Table S3 compares across pathogens regardless of physician diagnosis, but have the authors compared the clinical characteristics of the correctly diagnosed typhoid fever cases vs those that were incorrectly diagnosed? Did the cases that were incorrectly attributed have unusual clinical features? (the possibility also exists for co-infection, but that doesn't seem to have been assessed?) Although this is somewhat outside the scope of the authors' stated aims, it would be useful to understand factors that lead physicians to make the wrong diagnosis-- this could be accomplished by adding some columns to table S3 before the pooled 'All Salmonella' column

#### Our response:

The authors thank the reviewer for highlighting this point. We have added the correctly vs incorrectly diagnosed in Supplemental Table 7.

‒	Line 305-306: This sentence is unclear about the findings re diarrhea.

#### Our response:

The authors thank the reviewer for highlighting this point. The line has been revised based on reviewers’ suggestions. We have revised the wordings on line 371-2.

‒	Line 317-318: The meaning of this is unclear -- is it that the time from symptom onset until testing was similar (as in when the blood was drawn that was tested?) or the time delay between patient presentation and the availability of the laboratory results-- i think the former based on Table 2, but please clarify in the text.

#### Our response:

The authors thank the reviewer for highlighting this point. The text has been clarified as follows (lines 282-3):

*“TUBEX TF and Salmonella IgM and IgG testing showed little difference in the average number of days from symptom onset to specimen collection for testing.”*

‒	I think it would help with the flow of the text if the last paragraph of the results -- eg the description of Fig S3 were moved to after table 2. Then all the remaining results will only be concerned with confirmed typhoid (eg not including the probable typhoid).

#### Our response:

The authors thank the reviewer for highlighting this point. We have moved figure S3 to after table 2.

‒	Line 314-315: the text says 91 participants tested in clinically non-typhoid fever group. But the denominators in Fig 1 in the TUBEX >=4 column in the bottom section add up to 109-- what is the explanation for this difference?

#### Our response:

The authors thank the reviewer for highlighting this inconsistency. The correct number was 109 and has been revised in line 379.

‒	Similarly I am not sure who is included in the denominators in Table 3 for the TUBEX 134 confirmed non-Salmonella compared to the numbers presented in Fig 1. The control selection needs to be more clearly described (consider adding a section in the Methods after Case definitions).

#### Our response:

The authors thank the reviewer for highlighting this point. We have added the samples used for specificity analysis in lines 271-9 and notes section of Table 3.

‒	Table 3: for the IgG it says 49 had >=1.1 but Table S1 suggests that only 44 were positive by IgG (30 with IgM and IgG > 1.1 + 11 with IgG > 1.1, IgM negative + 3 with IgM seroconversion, IgG high -- is that correct?) . The methods section and Table S1 use >1.1 rather than >= 1.1 as in Table 3. Is this a typo? If not, use a consistent threshold definition throughout the manuscript.

#### Our response:

The authors thank the reviewer for highlighting this typo. The correct version is >1.1. We have corrected the statement in the manuscript.

‒	It would also be worth commenting somewhere in the text on whether there was any difference in the performance of the TUBEX for the probable Salmonella cases compared to the confirmed Salmonella-- eyeballing from Fig 1, it looks fairly similar.

#### Our response:

The authors thank the reviewer for highlighting this point. We have analyzed the difference between confirmed and probable enteric fever and the result is presented in Table S6.

***Minor comments***

‒	Line 273: Typhi should be capitalized

#### Our response:

The authors thank the reviewer for highlighting this error. The text has been revised and corrected to enteric fever.

‒	Table S3: Why is the total number of patients here 49 not 54?

#### Our response:

The authors thank the reviewer for highlighting this point. The table has been revised to S4 Table based on the main manuscript flow. *S.* Typhi was found in 39 cases, *S.* Paratyphi in 10, and *Salmonella* spp. in 5. The following note has been added to the bottom of the table:

*“Notes: •p-value <0.05. Among the 54 confirmed enteric fever cases observed in this study, 39 were S. Typhi, 10 were S. Paratyphi A, and 5 were non-speciated Salmonella spp. (excluded from this table).”*

‒	Line 338-340-- this is in the table, but specify that this is based on the ELISA result only from the acute sample, not including seroconversions.

#### Our response:

The authors thank the reviewer for highlighting this point. We have specified that ELISA was performed on acute samples in lines 412-4.

‒	Table 2: Consider shifting columns so that “Chikungunya” fits on one line; Probable Salmonella header is left aligned and the other headings are centered

#### Our response:

The authors thank the reviewer for highlighting this point. We have revised the Table.

‒	Figure S3: Recommend splitting this into a 2 panel vertical figure rather than overlaying the lines and bars and having 2 y-axes -- it will be easier to interpret

#### Our response:

The authors thank the reviewer for highlighting this point. We have split this figure into Figures S1 and S2.

--------------------

## Conclusions

### Reviewer #1:

‒	In conclusion, author recommend about the strict protocol for storage and process as per leaflet, however, author has not mentioned about the finding of problem of storage or process, neither author has discussed about this issue in detail. Thus, this conclusion is not supported by the information presented.

#### Our response:

The authors thank the reviewer for highlighting this point. The varied sensitivity between sites (stated in lines 410-2) showed the impact of processing to TUBEX TF sensitivity. We have also added more explanation in lines 496-8 and 499-501.

### Reviewer #2:

‒	I consider the conclusions supported by the data and the limitations have been described. The clinical and public health relevance of the results are addressed

### Reviewer #3:

‒	Lines 388-390: It is curious that the authors raise this limitation when it seems like they have access to the data to perform the analysis of/comparison to patients not diagnosed with typhoid fever.

#### Our response:

The authors thank the reviewer for highlighting this point. We have deleted this limitation. We have analyzed the clinical characteristics for both enteric and non-enteric fever cases and the result was described in Table S4.

‒	Line 432-433: I’m not sure if the ELISA kit used in this study is the same LPS, but Aiemjoy et al report on longitudinal kinetics of anti-S. Typhi LPS IgG. Doi: 10.1016/S2666-5247(22)00114-8

#### Our response:

The authors thank the reviewer for sharing this reference. The paper from Aiemjoy et al. describes an in-house ELISA using plates coated with *S.* Typhi LPS (2.5 µg/mL) purified from strain Ty21a (2.5 µg/mL). It is unclear if this is the same LPS used by the manufacturer of the ELISAs used in our study. However, this is a valuable reference that we have included in the discussion.

‒	Line 451-452: given this statement, it would be useful to provide (in the methods) details on who performed the TUBEX tests (hospital laboratory staff?) and whether they knew the provisional diagnosis of the patient.

#### Our response:

The authors thank the reviewer for highlighting this point. The following clarification has been added to the text (lines 180-2):

*“Specimens and accompanying clinical information, including provisional diagnosis, were provided to laboratory staff, and test results were immediately returned to the attending physician to inform patient care decisions.”*

‒	Lines 459-461: Recommend moving this information about the National requirement to test with TUBEX to the end of the Introduction because it helps provide context for the study aims.

#### Our response:

The authors thank the reviewer for highlighting this point. We have added more information on TUBEX in lines 131-2.

***Minor comments***

‒	Line 438: should read ...and the need to DO batch testing...

#### Our response:

The authors thank the reviewer for highlighting this point. The text has been revised based on reviewer’s suggestion.

‒	Line 469: Italicize Salmonella

#### Our response:

The authors thank the reviewer for highlighting this typo. The text has been revised according to reviewer’s suggestion.

--------------------

## Editorial and Data Presentation Modifications?

### Reviewer #1:

‒	None

### Reviewer #2:

‒	Outlined in methods and results

## Reviewer #3:

‒	Figure S2 should be included in the main body of the paper.

#### Our response:

The authors thank the reviewer for highlighting this point. We have added figure S2 into the main body (figure 3).

‒	Consider using term “low and middle income countries” or “typhoid affected countries” rather than the outdated term “developing countries.”

#### Our response:

The authors thank the reviewer for highlighting this point. We have revised the term developing countries into low- and middle-income countries (LMICs).

‒	Abstract methodology section mentions AFIRE study at eight “local hospitals.” Could a better description be used like “eight Indonesian hospitals” or “eight hospitals in [location]”?

#### Our response:

The authors thank the reviewer for highlighting this point. The revised text is in line 63.

‒	Introduction Lines 121 - 124 may want to also cite the more recent evaluation by Sapkota et al doi: 10.1128/jcm.01000-22

#### Our response:

The authors thank the reviewer for highlighting this point. We have added the result from Sapkota et al. in lines 134-5.

‒	Introduction: Line 130: Consider adding sentence describing mechanism of TUBEX, potentially moving the descriptive sentence from Lines 166-168.

#### Our response:

The authors thank the reviewer for highlighting this point. We have added more information on TUBEX in line 129.

--------------------

## Summary and General Comments

### Reviewer #1:

The study present the performance of TUBEX TF in detecting the Typhoidal Salmonella infection using the culture or PCR positive typhoid infection or other alternate etiology in existing health care delivery system and showed the difference in the real world performance in comparison to controlled study environment. This highlight the need of the high performing test kit for the diagnosis of the typhoidal Salmonella infection.

***Comment:***

- The author has used the typhoid fever, which is invasive bacterial infection caused by Salmonella Tyhi, where the result has shown both Salmonella Typhi and Paratyphi. It will be better to use the term Enteric fever or Typhoidal Salmonella infection to represent the both Salmonella Tyhpi and Paratyphi infection. (https://www.nature.com/articles/s41572-023-00480-z, <https://www.bmj.com/content/372/bmj.n437>)

#### Our response:

The authors thank the reviewer for highlighting this point. We have revised the manuscript using enteric fever.

### Reviewer #2:

The clinical diagnosis of enteric fever in endemic areas is difficult because the signs, symptoms and basic laboratory test results do not differentiate enteric fever from other infections such as dengue, chikungunya, rickettsisial infections and leptospirosis. Blood culture lacks sensitivity and is often unavailable. PCR on blood can be used but also lacks sensitivity. Ideally, a point of care test is required when the clinical decision about antibiotic use is made. TUBEX TF is a commercial test that is suggested for this purpose. It is a serological test that detects IgM antibodies to S. Typhi O:9 lipopolysaccharide antigen with a color score used to grade the response.

This retrospective analysis of the results of blood culture, blood PCR and TUBEX TF in a prospectively recruited group of patients with an acute febrile illness attending eight hospitals in Indonesia clearly illustrates these challenges.

The analysis of clinical symptoms and simple laboratory in the groups of patients with confirmed diagnoses for enteric fever and other infections demonstrates the overlap of symptoms and the lack of specific symptoms that distinguish each etiology. The low proportion of confirmed cases of enteric fever among those with a ‘clinical diagnosis’ of enteric fever and the finding of blood culture positive cases of enteric fever among those with a ‘clinical diagnosis’ of not enteric fever clearly illustrates the problem.

The performance of TUBEX TF for the diagnosis of enteric fever showed high sensitivity but very low specificity. There were significant levels of positivity for TUBEX TF in participants with negative blood culture and blood PCR and another microbiologically confirmed pathogen. This lack of diagnostic accuracy was more marked than has been reported in other published data on TUBEX TF. The variation in performance between different centres may be due to the subjectivity of grading the positivity of the result and that the test is a bit more complicated than a lateral flow test.

It is interesting that the specificity of the IgM and IgG ELISA appeared to be significantly better than the TUBEX TF test. However, it is not clear what antigen is used in the ELISA and this limits the interpretation of this result.

#### Our response:

The authors thank the reviewer for highlighting this point. We are also curious about this part. The insert kit only stated anti-lipopolysaccharide (LPS). We have asked the manufacturer about the antigen used in the ELISA kit, however, they can only provide membrane protein.

The report is generally clearly written and highlights the study limitations. An important limitation is that the various diagnostic tests used were not performed unselectively on all samples. It is a little difficult to follow the rationale to understand which patients had which tests conducted.

#### Our response:

The authors thank the reviewer for highlighting this point. We have added more information to the Methods section and rearranged the Results section to make it easier to read. We hoped that this revised version would be clearer and easier to interpret.

### Reviewer #3:

This manuscript describes the clinical features and performance of enteric fever diagnostics pulled from the AFIRE study in eight Indonesian hospitals. The study features a comprehensive set of clinical observations and laboratory diagnostics and presents an analysis of the sensitivity and specificity of the TUBEX-TF test as well as S. Typhi LPS IgM and IgG ELISA assay.

There is other literature evaluating the sub-optimal performance of the TUBEX but given the requirement for use by the Indonesian National Health Insurance system, there is value in publishing this study which finds very poor performance (and little diagnostic utility) of the TUBEX. The presentation of the results suffers from some lack of clarity in description which can make it difficult to follow. Additionally, due to lack of clearly described inclusion/exclusion criteria for each analysis, it was not possible to connect the numbers across the various tables and the numbers in the Tables need to be confirmed or require additional explanation as noted above.

#### Our response:

The authors thank the reviewer for highlighting this point. We have added more explanation to the Methods section and also rearranged and added more information to the Results section to make this manuscript clearer.

Some additional analyses may be warranted -- specifically a comparison of clinical characteristics of laboratory confirmed vs not typhoid fever by clinician diagnosis (this is partially presented). The authors have focused on presenting clinical features of those cases diagnosed (correctly and incorrectly) as typhoid fever; it would be useful to understand the converse.

The authors pool Typhi and Paratyphi cases, but the TUBEX test was designed for Typhi so it could be useful to conduct a sensitivity analysis (in supplementary material) of the ROC in only Typhi cases.

#### Our response:

The authors thank the reviewer for highlighting this point. We have analyzed the difference of TUBEX TF in *S.* Typhi and Paratyphi cases. The result is presented in supplemental Table 7.
