# Supplementary material for: Clinical characteristics of enteric fever and performance of TUBEX TF IgM test in Indonesian hospitals
Source: PLoS Negl Trop Dis. 2024 Jul 25;18(7):e0011848. doi: 10.1371/journal.pntd.0011848 (PMC11315288; doi:10.1371/journal.pntd.0011848)
Supplement: Attachment — Submitted filename: Point-to-point response.docx [file pntd.0011848.s013.docx]

PLOS Neglected Tropical Diseases

June 3^rd^, 2024

Dear Editor,

We thank you for allowing us to submit a revision of our manuscript (PNTD-S-23-02082) entitled “Clinical Characteristics of Typhoid Fever and Performance of TUBEX TF IgM Test in Indonesian Hospitals.”

We thank the reviewers for their reviews and comments, which helped us improve our manuscript. We have addressed all comments from the reviewers. Please find our point-by-point response to each comment below. You can find the references at the end of the document. Edits in the manuscript are given in track changes. All authors of this manuscript have seen and approved the content of the revised version.

On behalf of all authors,

Sincerely,

Bachti Alisjahbana

Faculty of Medicine

Universitas Padjadjaran

Bandung, Indonesia

# Response to Reviewers

Two additional comments noted by the editor:

1. The order of the specificities in the abstract (line 70) is reversed

#### Our response:

The authors thank the editor for highlighting this point. We have updated the text (line 70) as follows:

*“TUBEX TF demonstrated a sensitivity of 97.6% to 70.7% and specificity of 38.3% to 67.2% at cutoffs of 4 and 6, respectively.”*

2. The subtext of Table 3 should be corrected to something like test positive/confirmed positive for sensitivity and test negative/confirmed negative for specificity

#### Our response:

The authors thank the editor for highlighting this point. We have added the following clarifying text to the column headers of the table:

| **Sensitivity**  **Confirmed Enteric Fever Cases:**  **Number Positive / Number Tested**  **(%), [95% CI]** | **Specificity**  **Confirmed Non-Enteric Fever Cases:**  **Number Positive / Number Tested**  **(%), [95% CI]** |
| --- | --- |

We have also changed the subtext on Table 3 as follows:

*"The sensitivity analysis examined the number of confirmed enteric fever cases that were also tested by TUBEX TF (n = 41) or ELISA IgG/IgM (n = 54). The specificity analysis examined the number of non-enteric fever cases confirmed by their corresponding standard test (culture or PCR or IFA or MAT) that were also tested by TUBEX TF or ELISA IgG/IgM. TUBEX TF was tested in 134 cases including Rickettsia spp. (n = 44), Leptospira spp. (n = 16), Dengue (n = 62), and Chikungunya (n = 12). ELISA IgG was tested in 75 acute samples including Rickettsia spp. (n = 31), Leptospira spp. (n = 8), Dengue (n = 26), and Chikungunya (n = 10). ELISA IgM was tested in 111 acute samples including Rickettsia spp. (n = 42), Leptospira spp. (n = 12), Dengue (n = 47), and Chikungunya (n = 10)."*

## Methods

### Reviewer #1:

Objectives are clearly articulated but second objectives " evaluate performance of TUBEX TF" should be evaluate performance of TUBEX TF and Ig M and IgG ELISA as result in table 3 shows sensitivity and specificity of both against blood culture or PCR.

#### Our response:

The authors thank the reviewer for highlighting this point. We have updated the text (lines 144-5) as follows:

*“This study aims to both describe the clinical characteristics of enteric fever patients enrolled in a prospective observational cohort study of patients hospitalized with febrile illness across seven Indonesian cities, and to evaluate the performance of TUBEX TF and IgM and IgG ELISA serology in a real-world Indonesian hospital setting using blood culture and PCR for Salmonella as reference tests.”*

- sensitivity analysis done for independent variable should be separated from sensitivity analysis done for validation of the test

#### Our response:

The authors thank the reviewer for highlighting this point. We have updated the text as follows:

*“**TUBEX TF sensitivity was determined from all confirmed enteric fever patients who had TUBEX TF testing as part of their hospital evaluation. Two TUBEX TF score categories, ≥4 and ≥6, were assessed since the manufacturer recommends interpreting a score of 4 as weakly positive and 6-10 as positive (22). IgM and IgG ELISA sensitivities were determined from all confirmed enteric fever cases where specimens were available for testing at the reference laboratory. Each diagnostic test was additionally assessed with a receiver operating characteristic (ROC) curve, and the area under the curve (AUC) was calculated.*

*TUBEX TF specificity was determined from confirmed non-enteric fever cases where TUBEX TF testing was performed based on clinical suspicion. IgM and IgG ELISA specificities were determined from confirmed non-enteric fever cases where specimens were available for testing at the reference laboratory. Confirmed non-enteric fever cases in this analysis referred to cases that were found to be positive for Rickettsia spp., Dengue, Chikungunya, or Leptospira spp. by blood culture, PCR, IFA, and/or MAT. Non-enteric fever cases that were positive by serology only were excluded.*

*Three sensitivity analyses of diagnostic accuracy were performed: first, stratification by site to check for heterogeneity of diagnostic accuracy; second, stratification by age (<18 years versus ≥18 years) to assess whether patient age affected diagnostic accuracy; and third, restriction to only patients with both TUBEX TF or ELISA results to perform a direct comparison of tests.”*

## Results

### Reviewer #3:

- Figure S3 -- it would be good to include a legend that explains that the heavy solid lines are means (was a loess smoother used?)

#### Our response:

The authors thank the reviewer for highlighting this point. Yes, in this analysis we use loess smoother. We have updated the text (lines 750-3) as follows:

*“Notes: The figure shows IgM and IgG results for every visit for all subjects. The heavy lines represent the median values of IgM and IgG for all Salmonella cases (black lines), pediatric Salmonella cases (red lines), adult Salmonella cases (blue lines), and all non-Salmonella cases (green lines). A loess smoother was applied in each instance.”*

## Conclusions

### Reviewer #3:

-Line 490 -- I believe there is an extraneous period before the references (34,35)

#### Our response:

The authors thank the reviewer for highlighting this point. The line had been updated into line 486. We have deleted the period before (34,35).

-Line 510-512 -- I suggest moving the section on sensitivity before the paragraph on specificity (or integrating it). However, I don't think you are advocating for using a >=4 cutoff given the poor specificity, so you may want to consider this language a bit more.

#### Our response:

The authors thank the reviewer for highlighting this point. We have combined both paragraphs and revised the sentences. The revised statements are as follows:

*“The highest sensitivity (97.6%) was seen with TUBEX TF using a cut-off ≥4, but this was accompanied by the lowest specificity (38.8%). A cut-off score of ≥6 resulted in much higher, though still suboptimal, specificity (67.2%) at the expense of lower sensitivity (70.7%). Interestingly, our specificity results are lower than those previously reported (17, 36, 37). Over half of clinically diagnosed enteric fever cases with positive TUBEX TF results were confirmed attributable to non-Salmonella pathogens. The subjective nature of operator interpretation, suggested by the highly variable specificity across sites, may have contributed to an overestimation of enteric fever cases. In contrast, only 2 cases of clinically diagnosed enteric fever that were non-Salmonella were found to be positive by IgM ELISA. The better performance of S. Typhi IgM ELISA compared to TUBEX TF may reflect the subjectivity or cross-reactivity of TUBEX TF. If the observed performance were attributable to population characteristics, we would expect IgM ELISA to perform similarly to TUBEX TF. Moreover, sensitivity and specificity of TUBEX TF varied between sites, unlike ELISA performance. This result emphasizes the subjectivity of grading positive results and the difficulties of this test compared to lateral flow assays. Performance of TUBEX TF might be improved with better standardization of reagent storage and assay techniques (38). Incorporating TUBEX TF into a clinical algorithm to diagnose enteric fever may also improve its utility so long as it is not the sole diagnostic test (39).”*

-- Line 559 -- should read ...to study TUBEX TF in A controlled setting.

#### Our response:

The authors thank the reviewer for highlighting this point. The line had been updated into line 553 and ee have added “a” in line 553.

## Summary and General Comments

### Reviewer #3:

In a few places the replacement of typhoid with enteric fever is incomplete (line 58, 119)

#### Our response:

The authors thank the reviewer for highlighting this point. We have revised lines 58 and 119, which are now written as “enteric fever”.

Line 92: should read ...enteric fever cases HAS been.. (the use of clinical data...has been considered.)

#### Our response:

The authors thank the reviewer for highlighting this point. We have revised *“have”* in line 92 with *“has”*.

-Line 136-138 I think this sentence should precede the sentence about the Sapkota manuscript because I think it is describing the studies included in the meta-analysis referenced in line 133?

#### Our response:

The authors thank the reviewer for highlighting this point. We have moved the sentence “The studies included were designed to assess TUBEX TF, and therefore tended to overestimate the accuracy of test results” from lines 136-8 to lines 135-6. The sentences now read:

*“A meta-analysis showed an average sensitivity of 78% (95% CI 71% to 85%) and specificity of 87% (95%CI 82% to 91%) for TUBEX TF with a cut-off ≥ 3.* *The studies included were designed to assess TUBEX TF and therefore tended to overestimate the accuracy of test results (16). A study from Sapkota et al. also revealed that TUBEX TF had a sensitivity and specificity of 60.6% and 94.0%, respectively (20).”*
